# Structure and dynamics in the lithium solvation shell of nonaqueous electrolytes

**DOI:** 10.1038/s41598-019-42050-y

**Published:** 2019-04-03

**Authors:** Sungho Han

**Affiliations:** CAE Group, AMD Lab, AI&SW Research Center, Samsung Advanced Institute of Technology, Suwon, Gyeonggi 16678 Korea

## Abstract

The solvation of a lithium ion has been of great importance to understand the structure and dynamics of electrolytes. In mixed electrolytes of cyclic and linear carbonates, the lithium solvation structure and the exchange dynamics of solvents strongly depend on the mixture ratio of solvents, providing a connection of the rigidity of the lithium solvation shell with the solvent composition in the shell. Here we study the dynamical properties of solvents in the solvation sheath of a lithium ion for various solvent mixture ratios via molecular dynamics simulations. Our results demonstrate that the exchange dynamics of solvents exhibits a non-monotonic behavior with a change in the mixture ratio, which keeps preserved on both short and long time scales. As the fraction of cyclic carbonate increases, we find that the structural properties of cyclic and linear carbonates binding to a lithium ion show different responses to a change in the fraction. Furthermore, we find that the rotational dynamics of cyclic carbonate is relatively insensitive to the mixture ratio in contrast to the rotational dynamics of linear carbonate. Our results further present that an anion shows different properties in structure and dynamics from solvents upon changing the mixture ratio of solvents.

## Introduction

An electrolyte is one of indispensable components of lithium ion batteries^1–5^. It serves as media for lithium ions to move back and forth between cathode and anode during charging and discharging operations^1,5^. The properties required for being good electrolytes of lithium ion batteries includes the good solubility of salt, the good fluidity for the ionic transport and the good stability from any reactions during the battery operation. However, one solvent type in nonaqueous electrolytes cannot satisfy all requirements of electrolytes. Generally, solvents with high dielectric constants present the good solubility of salt but they invoke the high viscosity of electrolytes due to their polar nature, generating the slow transport of Li^+^ ions. For solvents with low dielectric constants, on the other hand, they provide the good environments for the fast transport of ions but easily induce the undesirable ion-pairing of cations and anions due to their low solubility. For commercial lithium ion batteries, as a result, the mixed electrolytes consisting of cyclic and linear carbonates such as ethylene carbonate (EC) and dimethyl carbonate (DMC) have generally been used to enhance both the solubility of salt and the mobility of ions, simultaneously. If the fraction of cyclic carbonate in the electrolyte increases, the solubility will also increase but the mobility of ions will undesirably decrease in general. In contrast, if the fraction of linear carbonate increases, the mobility of ions will be improved but the solubility will be worse. In the mixed electrolytes, thus, finding an optimal mixture ratio of solvents has been of great interest to improve the performance of lithium ion batteries.

For the mixed electrolytes of lithium ion batteries, it has been long believed that solvents with high and low dielectric constants, such as EC (*ε* ∼ 90 at 40 °C) and DMC (*ε* ∼ 3.1 at 25 °C), play distinct roles in the electrolyte^1^. Preferentially, EC participates in solvating a Li^+^ ion and contributes to form lithium-solvents complexes^6,7^. On the other hand, DMC serves as media for the Li^+^ ion-solvents complexes to transport in the electrolyte. Recently, however, many studies have shown that both types of solvents are able to actively participate in forming the lithium solvation sheath and the main factor to determine the composition of the lithium solvation shell is simply the mixture ratio between them, although their dielectric constants show a large difference in magnitude^8–15^. The structure of the lithium solvation sheath has been considered to be crucial for forming the protective film on the electrodes, known as a solid electrolyte interphase (SEI), because the solvents in the solvation sheath predominantly participate in forming the SEI by decomposition^16–18^. The mixture ratio of binary solvents further affect the ionic conductivity, showing a non-monotonic dependence of the ionic conductivity on the mixture ratio of solvents^1,19^. The non-monotonic behavior in the ionic conductivity is ascribed to a competition between the viscosity of the electrolyte and the ion-pairing of cations and anions. This non-monotonic behavior of the ionic conductivity has also been found in its dependence on the salt concentration^1,19^.

Generally, one considers the rigidity of the solvation shell of ions in electrolytes to be critical for the mobility of ions^20–26^. As the rigidity of the solvation shell increases, the ionic transport slows down due to an increase in the drag against the motion of lithium-solvents complexes^15^. The rigidity of the solvation shell can be characterized by the measure of the residence time of solvents within the solvation shell, which presents how easily the solvation structure can be broken. Hence, the rigidity of the solvation shell is closely related with the exchange dynamics of solvents in the solvation shell – in other words, how long the solvents can reside in the solvation shell^15,26–28^. The faster exchange dynamics of solvents invokes the weaker rigidity of the solvation shell due to the weaker bonding with a Li^+^ ion. Obviously, the solvation dynamics has a close connection with the structure of the lithium solvation shell^15^. Since the solvation structure depends on the mixture ratio of solvents, the solvation dynamics would be affected by the mixture ratio as well. Thus, finding the relation between the solvation dynamics and the mixture ratio of solvents would be of significance to broaden our understanding of electrolytes and design them suitable for the future lithium ion batteries.

In this work, we investigate the dynamics in the lithium solvation shell of nonaqueous electrolytes consisting of 1 M lithium hexafluorophosphate (LiPF_6_) with binary solvents of EC and DMC as a function of the solvent mixture ratio at the temperature of *T* = 300 K. We examine the six different mixture ratios from EC:DMC = 10%:90% up to 60%:40%. For simplicity, we will denote the mixture ratio of binary solvents as only the EC fraction, χ_EC_, throughout this work.

## Results and Discussion

### Solvation dynamics in the lithium solvation shell

**Figure 1:**
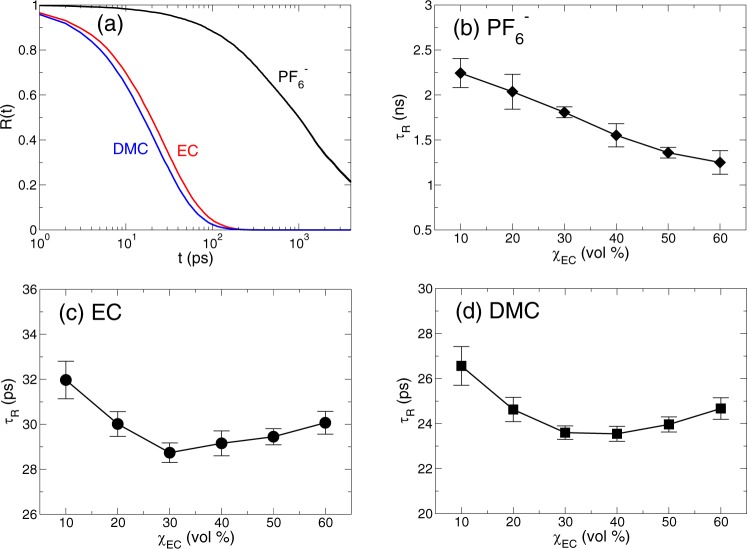
The exchange dynamics in the lithium solvation shell on a short time scale. (**a**) The residence time distributions *R*(*t*) of a ${{\rm{PF}}}_{6}^{-}$ ion, EC and DMC as a function of time *t* for the EC fraction of χ_EC_ = 30%. Next, shown is the characteristic residence time *τ*_*R*_ of (**b**) ${{\rm{PF}}}_{6}^{-}$, (**c**) EC and (**d**) DMC as a function of χ_EC_. Whereas ${\tau }_{R}^{{{\rm{PF}}}_{6}^{-}}$ monotonically decreases with increasing χ_EC_, *τ*_*R*_ for the two solvents, EC and DMC, exhibits a non-monotonic behavior with respect to χ_EC_. It shows a minimum around the value of χ_EC_ between 30% and 40%.

First, we consider how long solvents are able to reside in the first solvation shell of a Li^+^ ion as a function of χ_EC_. For the sake of it, we examine the slow and fast solvation dynamics of solvents in the first solvation shell of a Li^+^ ion. The reason we consider two different solvation dynamics is that they occur on different time scales and they are based on the different underlying mechanisms^15,28^. First of all, we define the first solvation shell of a Li^+^ ion as the first plateau in the cumulative coordination number *n*(*r*)^15,28^, as we will see later. In this definition, the first solvation shell of a Li^+^ ion is defined as a circle centered at a Li^+^ ion with a radius of 0.3 nm for a carbonyl oxygen atom O_*c*_ of EC and DMC and a circle with a radius of 0.45 nm for a central P atom of a ${{\rm{PF}}}_{6}^{-}$ ion^15^. For the fast solvation dynamics, we define the residence time distribution *R*(*t*) as^15,28–30^

1 $$R(t)\equiv \langle {\rm{\Theta }}({t}_{b}-t)\rangle ,$$

where Θ(*t*) is the Heaviside step function, *t*_*b*_ is the first-passage time for a solvent to be dissociated from the lithium solvation shell and 〈…〉 represents an ensemble average. In this definition of *R*(*t*), we consider only the intact bonding of a solvent with a Li^+^ ion for a given time interval. The fast solvation dynamics is known to be closely related with the motions occurred on a short time scale, such as the thermal fluctuation^15,28,31^. In Fig. 1(a), we present *R*(*t*) of EC, DMC, and ${{\rm{PF}}}_{6}^{-}$ as a function of time *t* at the EC fraction of χ_EC_ = 30%. It shows that *R*_*DMC*_(*t*) decays faster than *R*_*EC*_(*t*)^32^ and ${R}_{P{F}_{6}^{-}}(t)$ decays much slower than both *R*_*EC*_(*t*) and *R*_*DMC*_(*t*). It indicates that by the thermal fluctuation the two solvents can escape the lithium solvation shell much faster than an anion due to the strong Coulombic interaction of the anion with a cation. As for both solvents, DMC forms the weaker bonding with a Li^+^ ion than EC, so that *R*_*DMC*_(*t*) decays faster than *R*_*EC*_(*t*)^15,32^. Note that those decaying behaviors on a short time scale are valid for all χ_EC_s we investigated.

To characterize the temporal behavior of *R*(*t*) in terms of a single value, we define the characteristic residence time *τ*_*R*_ as the time required for *R*(*t*) to decay by a factor of *e*^15,28,30^. In Fig. 1(b–d), we present *τ*_*R*_ of ${{\rm{PF}}}_{6}^{-}$, EC and DMC as a function of χ_EC_. Our results show that the exchange dynamics of EC and DMC occurs on the time scale of tens of picoseconds, whereas the exchange dynamics of ${{\rm{PF}}}_{6}^{-}$ occurs in a few nanoseconds. The direct observations on the solvation dynamics have been limited by the experimental difficulties due to the nature of ultrafast dynamics. However, a recent experiment using the coherent two-dimensional infrared spectroscopy has shown that the residence of a solvent in the solvation shell of a Li^+^ ion has indeed a finite lifetime and the fast solvation dynamics occurs on the time scale of tens of picoseconds^31^. Our results of the fast solvation dynamics in tens of picoseconds are in good agreement with the experimental results^31^. The behaviors of ${\tau }_{R}^{{\rm{EC}}}$ and ${\tau }_{R}^{{\rm{DMC}}}$ in terms of χ_EC_ are quite different from ${\tau }_{R}^{{{\rm{PF}}}_{6}^{-}}$ which decreases monotonically with the increasing χ_EC_. We find that both ${\tau }_{R}^{{\rm{EC}}}$ and ${\tau }_{R}^{{\rm{DMC}}}$ exhibit non-monotonic behaviors as a function of χ_EC_. As χ_EC_ increases to 30%, both ${\tau }_{R}^{{\rm{EC}}}$ and ${\tau }_{R}^{{\rm{DMC}}}$ decrease the same as in ${\tau }_{R}^{{{\rm{PF}}}_{6}^{-}}$. When χ_EC_ further increases, however, we find that ${\tau }_{R}^{{\rm{EC}}}$ and ${\tau }_{R}^{{\rm{DMC}}}$ now increase, showing the minimum in ${\tau }_{R}^{{\rm{EC}}}$ and ${\tau }_{R}^{{\rm{DMC}}}$ between χ_EC_ = 30% and 40%.

**Figure 2:**
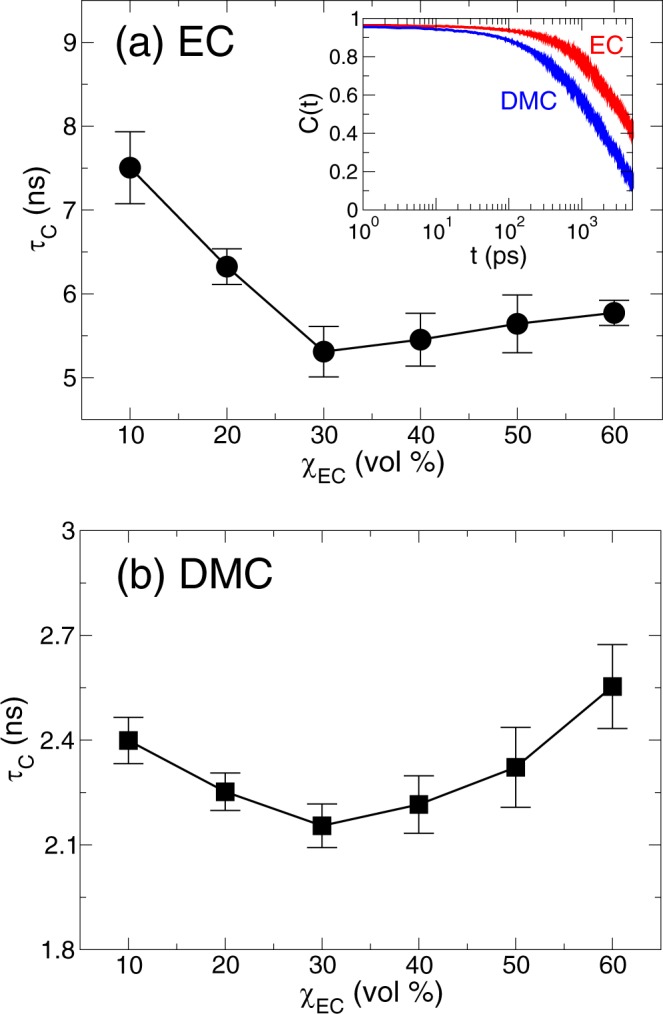
The exchange dynamics in the lithium solvation shell on a long time scale. The characteristic residence correlation times *τ*_*C*_ of (**a**) EC and (**b**) DMC as a function of χ_EC_. Inset: the residence correlation time distributions *C*(*t*) of EC and DMC as a function of time *t* in a semi-log plot at the EC fraction of χ_EC_ = 30%.

We further find the similar non-monotonic behaviors in the slow solvation dynamics in terms of χ_EC_. We describe the slow solvation dynamics using the residence correlation time distribution *C*(*t*) defined as^15,28,30^

2 $$C(t)\equiv \frac{\langle h(t)\cdot h\mathrm{(0)}\rangle }{\langle h\mathrm{(0)}\cdot h\mathrm{(0)}\rangle },$$

where *h*(*t*) is unity when a solvent is within the first solvation shell of a Li^+^ ion and *h*(*t*) is zero, otherwise. *C*(*t*) indicates the conditional probability that a bonding with a Li^+^ ion remains intact at time *t*, given it was intact at time *t* = 0. In contrast to *R*(*t*), *C*(*t*) does not consider any breaking of the bond at intermittent times between time *t* = 0 and *t*. *C*(*t*) is closely connected with the motions on a long time scale, such as the diffusive motions. In the inset of Fig. 2(a), we present *C*(*t*) of EC and DMC at the EC fraction of χ_EC_ = 30%. *C*_EC_(*t*) decays slower than *C*_DMC_(*t*), indicating the slower diffusion of EC than DMC. To characterize the temporal behavior of *C*(*t*) in terms of a single value, we also define the characteristic correlation time *τ*_*C*_ in the same way as in *τ*_*R*_. In Fig. 2, we present ${\tau }_{C}^{{\rm{EC}}}$ and ${\tau }_{C}^{{\rm{DMC}}}$ as a function of χ_EC_. We find that ${\tau }_{C}^{{\rm{EC}}}$ and ${\tau }_{C}^{{\rm{DMC}}}$ exhibit the same non-monotonic behaviors as in ${\tau }_{R}^{{\rm{EC}}}$ and ${\tau }_{R}^{{\rm{DMC}}}$ with respect to χ_EC_. It indicates that the dynamic characteristic features of the solvation dynamics on a short time scale keep preserved on a long time scale. Both ${\tau }_{C}^{{\rm{EC}}}$ and ${\tau }_{C}^{{\rm{DMC}}}$ exhibit the minimum around at χ_EC_ = 30%.

The non-monotonic exchange dynamics of solvents is ascribed to various and complex factors such as the composition of solvents in the lithium solvation shell, an intensity of the bonding of solvents with a Li^+^ ion, the translational and rotational motions of solvents, the interaction between solvents within the lithium solvation shell, the interaction between solvents inside and outside the solvation shell and the position of solvents in the solvation shell, etc. Investigation of only one or two factors might be insufficient to reveal the full underlying mechanism for the non-monotonic exchange dynamics. In despite of it, however, it would be worthy of exploring how some of the factors described the above would be connected with the solvation dynamics.

### Structure of the lithium solvation shell

**Figure 3:**
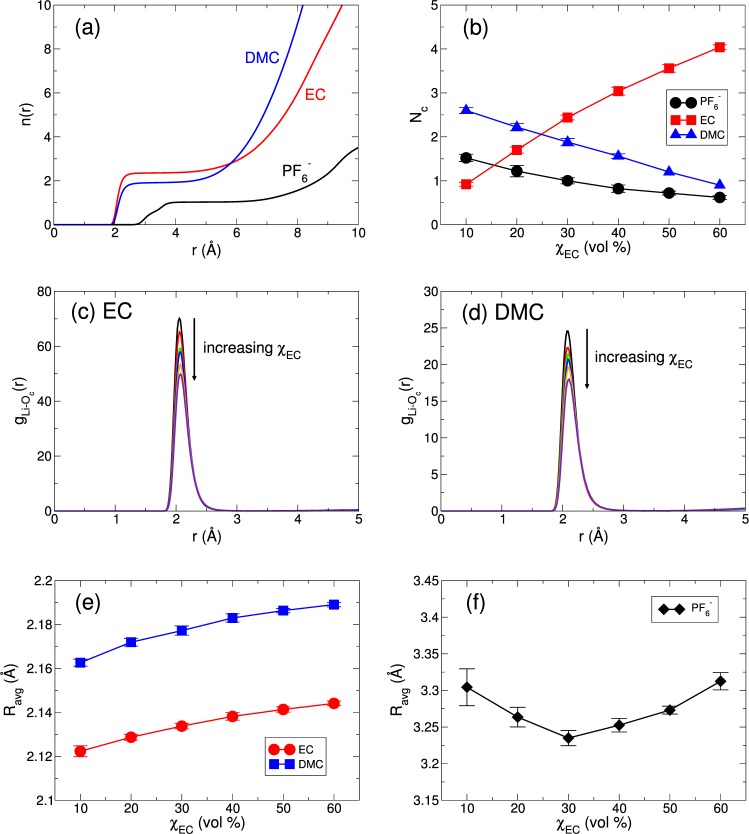
The structure of the first solvation shell of a Li^+^ ion. (**a**) The cumulative coordination number *n*(*r*) as a function of distance *r* at the EC fraction of χ_EC_ = 30%. (**b**) The solvation number *N*_*c*_ in the first solvation shell of a Li^+^ ion. The radial distribution function ${g}_{L{i}^{+} \mbox{-} {O}_{c}}$ between a Li^+^ ion and a carbonyl oxygen atom of (**c**) EC and (**d**) DMC as a function of distance *r*. The first peak in ${g}_{L{i}^{+} \mbox{-} {O}_{c}}$ is positioned at *r* = 2.06 Å for EC and 2.09 Å for DMC, respectively. (**e**) The average distance *R*_avg_ of the carbonyl oxygen atom from a Li^+^ ion in the first solvation shell for EC and DMC as a function of χ_EC_, respectively. (**f**) The average distance *R*_avg_ of the phosphorus atom of ${{\rm{PF}}}_{6}^{-}$ from a Li^+^ ion as a function of χ_EC_.

Next, we investigate the structure of the lithium solvation shell as a function of χ_EC_. First, we calculate the cumulative coordination number *n*(*r*) defined as

3 $$n(r)\equiv 4\pi \rho {\int }_{0}^{r}{r^{\prime} }^{2}g(r^{\prime} )dr^{\prime} ,$$

where *g*(*r*) is the radial distribution function (RDF). In Fig. 3(a), we present *n*(*r*) of three components of the electrolyte as a function of distance *r* from a Li^+^ ion at the EC fraction of χ_EC_ = 30%. To calculate *n*(*r*), we use the positions of the carbonyl oxygen O_*c*_ atom for EC and DMC and the P atom for ${{\rm{PF}}}_{6}^{-}$. We find one plateau i*n n*(*r*) for all three components, indicating that there is one solvation shell of a Li^+^ ion. We define the first plateau i*n n*(*r*) as the first solvation shell of a Li^+^ ion and the value of *n*(*r*) at the first plateau as the solvation number *N*_*c*_ in the first solvation shell of a Li^+^ ion^15^. In Fig. 3(b), we present the solvation number *N*_*c*_ as a function of χ_EC_. As for χ_EC_ = 10%, ${N}_{c}^{{\rm{DMC}}}$ (=2.60) is larger than ${N}_{c}^{{\rm{EC}}}$ (=0.92), showing that a Li^+^ ion is solvated mostly by DMC. When χ_EC_ further increases, ${N}_{c}^{{\rm{EC}}}$ increases and ${N}_{c}^{{\rm{DMC}}}$ decreases, resulting in that the majority of the first solvation shell of a Li^+^ ion becomes EC instead of DMC. We note that the total solvation number ${N}_{c}^{{\rm{total}}}(={N}_{c}^{{{\rm{PF}}}_{6}^{-}}+{N}_{c}^{{\rm{EC}}}+{N}_{c}^{{\rm{DMC}}})$ of the first solvation shell of a Li^+^ ion increases from ${N}_{c}^{{\rm{total}}}=5.1$ at χ_EC_ = 10% to ${N}_{c}^{{\rm{total}}}=5.6$ at χ_EC_ = 60%. Thus, as χ_EC_ increases, the lithium-solvents complex becomes larger and heavier.

We further examine the structure between a Li^+^ ion and the two solvents. We calculate ${g}_{{\rm{Li}} \mbox{-} {{\rm{O}}}_{{\rm{c}}}}$ between a Li^+^ ion and the carbonyl oxygen atom O_*c*_ of EC and DMC^33–35^. The position of the first peak in ${g}_{{\rm{Li}} \mbox{-} {{\rm{O}}}_{{\rm{c}}}}$ is not influenced by the change in χ_EC_, but the intensity of the first peak decreases as χ_EC_ increases. Even though the position of the first peak in ${g}_{{\rm{Li}} \mbox{-} {{\rm{O}}}_{{\rm{c}}}}$ does not change, the distribution of the O_*c*_ positions of EC and DMC in the lithium solvation shell could be affected by the change in χ_EC_ due to the change in the shape of the first peak of ${g}_{{\rm{Li}} \mbox{-} {{\rm{O}}}_{{\rm{c}}}}$. To see the effect of χ_EC_ on a distance between solvents and a Li^+^ ion in the solvation shell, we further calculate the binding distance, that is, the average distance *R*_avg_ between a Li^+^ ion and the carbonyl oxygen atom O_*c*_ for EC and DMC within the first solvation shell of a Li^+^ ion. In Fig. 3(e), we present the average distance *R*_avg_ of EC and DMC as a function of χ_EC_. It shows that EC is generally located to a Li^+^ ion closer than DMC. For all χ_EC_s, ${R}_{{\rm{avg}}}^{{\rm{EC}}}$ is smaller than ${R}_{{\rm{avg}}}^{{\rm{DMC}}}$ by the same value of ${\rm{\Delta }}{R}_{{\rm{avg}}}(\,\equiv \,{R}_{{\rm{avg}}}^{{\rm{DMC}}}-{R}_{{\rm{avg}}}^{{\rm{EC}}})\sim 0.04$ Å. Even though this difference in Δ*R*_avg_ is too small, it appears consistently over the whole range of χ_EC_ we investigated. As χ_EC_ increases, both ${R}_{{\rm{avg}}}^{{\rm{EC}}}$ and ${R}_{{\rm{avg}}}^{{\rm{DMC}}}$ gradually increase. It indicates the increasing size of the first solvation shell of a Li^+^ ion with the increasing χ_EC_, which is directly related with the increasing size of the lithium-solvents complex. We find that the average position ${R}_{{\rm{avg}}}^{{{\rm{PF}}}_{6}^{-}}$ of a ${{\rm{PF}}}_{6}^{-}$ ion in the first solvation shell of a Li^+^ ion shows a non-monotonic behavior with respect to χ_EC_. Figure 3(f) shows the minimum in ${R}_{{\rm{avg}}}^{{{\rm{PF}}}_{6}^{-}}$ around χ_EC_ = 30%.

**Figure 4:**
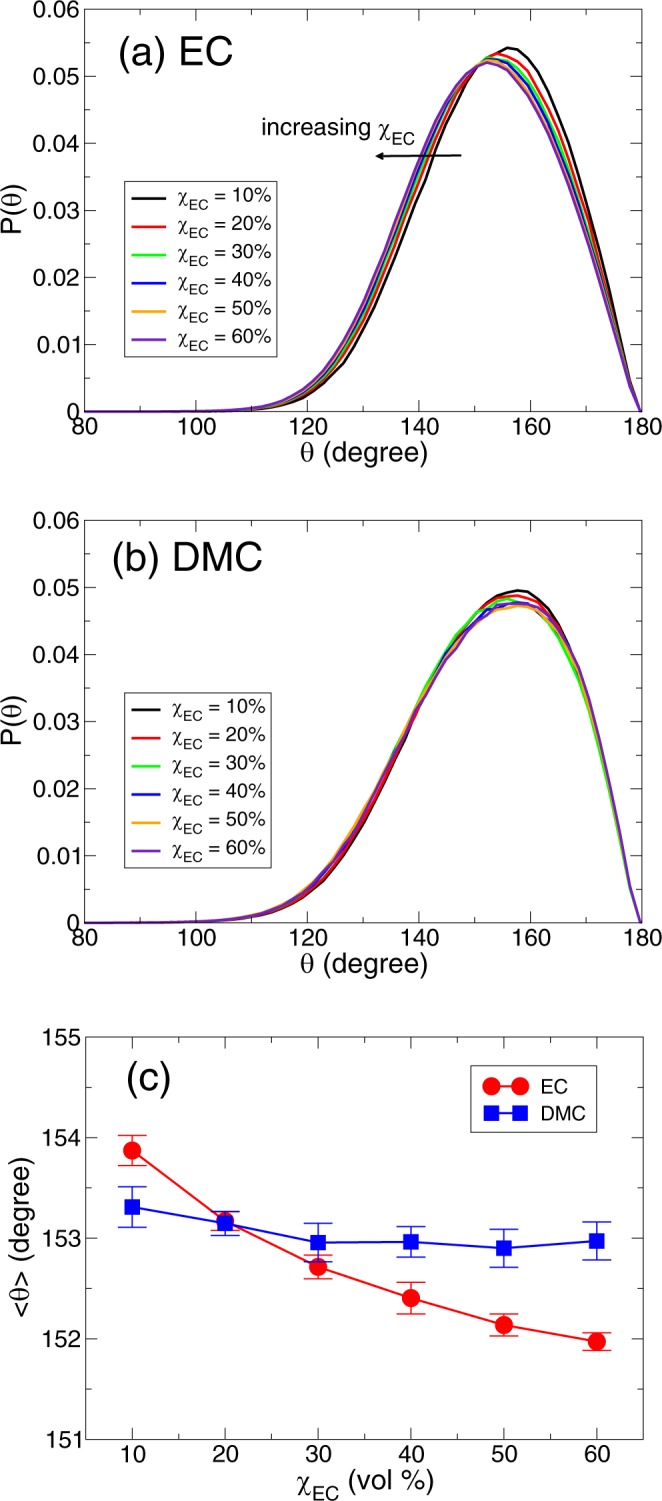
The binding direction of solvents with a Li^+^ ion. The angle distribution *P*(*θ*) for (**a**) EC and (**b**) DMC. (**c**) The averaged value 〈*θ*〉 of an angle *θ* between Li^+^ … O_*c*_ and O_*c*_=C of the carbonyl group of EC and DMC as a function of χ_EC_.

In addition to the average binding distance of the solvents, we consider the binding direction of EC and DMC with a Li^+^ ion to fully understand the nature of the lithium solvation structure as a function of χ_EC_^36^. Specifically, we investigate the distribution *P*(*θ*) of a binding angle *θ* between a Li^+^ ion and the carbonyl group of EC and DMC for various χ_EC_s. Here we consider an angle *θ* ≡ ∠Li^+^ O_*c*_ C, where O_*c*_=C is the carbonyl group of EC and DMC. In Fig. 4(a,b), we present *P*(*θ*) of EC and DMC. For EC, the maximum value in *P*(*θ*) occurs at ${\theta }_{{\rm{EC}}}^{{\rm{\max }}}\simeq {156}^{\circ }$ at χ_EC_ = 10% and it gradually decreases to ${\theta }_{{\rm{EC}}}^{{\rm{\max }}}\simeq {152}^{\circ }$ at χ_EC_ = 60%. For DMC, the maximum occurs at ${\theta }_{{\rm{DMC}}}^{{\rm{\max }}}\simeq {158}^{\circ }$ at χ_EC_ = 10% and it seems not to change upon increasing χ_EC_. For both solvents, the three atoms of Li, O_*c*_ and C tend to be slightly off a straight line^25,37,38^. Whereas *P*_*EC*_(*θ*) shows a shift toward the smaller angle upon changing χ_EC_, *P*_DMC_(*θ*) for all χ_EC_s does not change the shape of the curve. From the calculation of the average angle, $\langle \theta \rangle [\equiv \int \theta P(\theta )d\theta /\int P(\theta )d\theta ]$, we find that 〈*θ*_EC_〉 decreases upon increasing χ_EC_. In contrast, 〈*θ*_DMC_〉 is relatively insensitive to a change in χ_EC_. Note that the change Δ〈*θ*_EC_〉 in the average angle is quite small (${\rm{\Delta }}\langle {\theta }_{EC}\rangle \sim {1.9}^{\circ }$ between χ_EC_ = 10% and 60%).

### The translational and rotational dynamics of solvents

To examine how the solvation dynamics of a Li^+^ ion is related with the motions of solvents, we consider the translational and rotational dynamics of EC and DMC. For the translational motion, we calculate the translational mean square displacement (TMSD)^15,28,29,39–42^,

4 $$\langle {\rm{\Delta }}{\overrightarrow{r}}^{2}(t)\rangle \equiv \langle \frac{1}{N}\sum _{i=1}^{N}{[{\overrightarrow{r}}_{i}(t)-{\overrightarrow{r}}_{i}\mathrm{(0)]}}^{2}\rangle .$$

From the TMSD, we can calculate the translational diffusion constant *D*_*T*_ via the relation of

5 $$2d{D}_{T}t=\mathop{\mathrm{lim}}\limits_{t\to \infty }\langle {\rm{\Delta }}{\overrightarrow{r}}^{2}(t)\rangle ,$$

where *d* is the dimensionality of the system. To obtain an expression of the rotational mean square displacement (RMSD)^28,43^, we first define the vector $\overrightarrow{H}(t)\equiv \overrightarrow{C{O}_{c}}$ of the carbonyl group of EC and DMC. For a time interval *δt*, the vector $\overrightarrow{H}$ spans the angle $\delta \phi \equiv {\cos }^{-1}[\overrightarrow{H}(t+\delta t)\cdot \overrightarrow{H}(t)]$. An angle vector $\delta \overrightarrow{\varphi }$ is to be that the magnitude is $|\delta \overrightarrow{\varphi }(t)|\equiv \delta \phi$ and the direction is given by $\overrightarrow{H}(t)\times \overrightarrow{H}(t+\delta t)$. Finally, we obtain the angle vector $\overrightarrow{\varphi }(t)$ by summing $\delta \overrightarrow{\omega }(t)(\,\equiv \,\delta \overrightarrow{\varphi }(t)/\delta t)$ over time *t*,

6 $$\overrightarrow{\varphi }(t)={\int }_{0}^{t}dt^{\prime} \delta \overrightarrow{\omega }(t^{\prime} \mathrm{)}.$$

Now we are able to define the RMSD similar to the TMSD,

7 $$\langle {\rm{\Delta }}{\overrightarrow{\varphi }}^{2}(t)\rangle \equiv \langle \frac{1}{N}\sum _{i\mathrm{=1}}^{N}{[{\overrightarrow{\varphi }}_{i}(t)-{\overrightarrow{\varphi }}_{i}\mathrm{(0)]}}^{2}\rangle .$$

From the RMSD, we similarly calculate the rotational diffusion constant *D*_*R*_ via the relation^28,43^ of

8 $$4{D}_{R}t=\mathop{\mathrm{lim}}\limits_{t\to \infty }\langle {\rm{\Delta }}{\overrightarrow{\varphi }}^{2}(t)\rangle .$$

**Figure 5:**
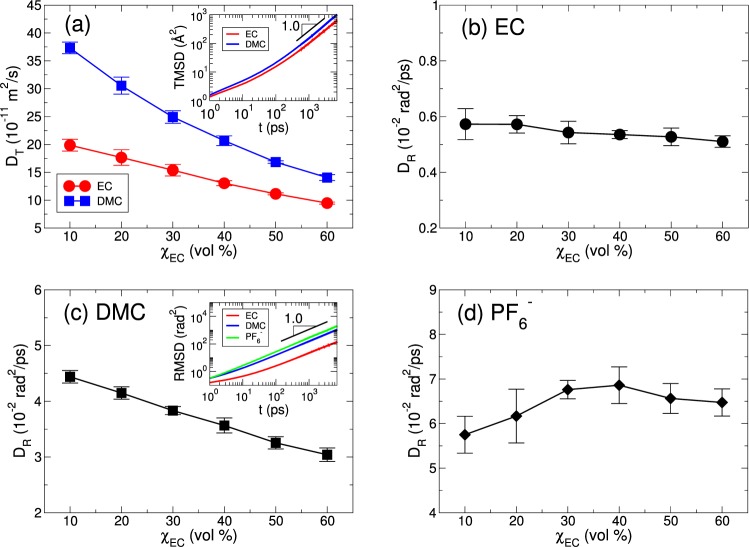
The translational and rotational diffusion constants. (**a**) The translational diffusion constants *D*_*T*_ of EC and DMC as a function of χ_EC_. Inset: the translational mean square displacements (TMSDs) of EC and DMC at χ_EC_ = 30% in a log-log plot, showing the diffusive regime (TMSD ∝ *t*) in the long time limit. The rotational diffusion constants *D*_*R*_ of (**b**) EC, (**c**) DMC and (**d**) ${{\rm{PF}}}_{6}^{-}$ as a function of χ_EC_. To calculate *D*_*R*_, we use a vector connecting the carbon atom with the oxygen atom in the carbonyl group (C=O_*c*_) for both EC and DMC. Inset in (**c**): the rotational mean square displacements (RMSDs) of EC, DMC and ${{\rm{PF}}}_{6}^{-}$ at χ_EC_ = 30% in a log-log plot, showing the diffusive regime (RMSD ∝ *t*) in the long time limit.

In Fig. 5, we present the translational diffusion constant *D*_*T*_ and the rotational diffusion constant *D*_*R*_ of EC and DMC as a function of χ_EC_. For the translational dynamics, both ${D}_{T}^{{\rm{EC}}}$ and ${D}_{T}^{{\rm{DMC}}}$ monotonically decrease as χ_EC_ increases^23,44^. Since EC has the much larger dielectric constant *ε* than DMC, the increase in χ_EC_ entails the increase in the viscosity of the electrolyte, so that the translational dynamics in the electrolyte becomes slower^1,5,15,45^. On the other hand, the rotational dynamics of EC, DMC and ${{\rm{PF}}}_{6}^{-}$ is different from the translational dynamics in terms of χ_EC_. In Fig. 5(b and c), we present ${D}_{R}^{{\rm{EC}}}$ and ${D}_{R}^{{\rm{DMC}}}$ as a function of χ_EC_. ${D}_{R}^{{\rm{DMC}}}$ decreases upon increasing *χ*_*EC*_, but ${D}_{R}^{{\rm{EC}}}$ shows a behavior nearly insensitive to χ_EC_. For EC, a difference in ${D}_{R}^{{\rm{EC}}}$ between χ_EC_ = 10% and 60% is ${\rm{\Delta }}{D}_{R}^{{\rm{EC}}}\,\sim \,$0.1 × 10^−2^ (rad^2^/ps). For DMC, ${\rm{\Delta }}{D}_{R}^{{\rm{DMC}}}$ is around 1.5 × 10^−2^ (rad^2^/ps), indicating that the effect of χ_EC_ on the rotational dynamics of EC is very weak. The difference in the rotational dynamics of EC and DMC comes from various factors. As mentioned before, the dielectric constant *ε* of EC (*ε* ∼ 90 at 40°) is much larger than DMC (*ε* ∼ 3.1 at 25°), so that it causes the bigger drag against a rotational motion for EC than DMC. The carbonyl oxygen atom O_*c*_ of EC and DMC forms a bond with a Li^+^ ion but the intensity of the bonding is different for EC and DMC. As shown in Figs 1 and 2, the residence time of EC within the lithium solvation shell is always longer than DMC on both short and long time scales. It causes the more drag against the rotational motion of EC than DMC. In addition, the molecular structures of EC and DMC also cause the difference in the rotational dynamics such that the carbonyl group of EC needs more energy to rotate than one of DMC, because the moment of inertia about the rotational axis of EC is bigger than DMC. Those conditions result in the fact that the rotational dynamics of EC is slower than DMC by a factor of 7∼8.

The rotational dynamics of a ${{\rm{PF}}}_{6}^{-}$ ion exhibits an interesting feature, as shown in Fig. 5(d). Due to the strong Coulombic interaction between cations and anions, the translational dynamics of ${{\rm{PF}}}_{6}^{-}$ is known to be much slower than EC and DMC^15^. Even though the residence time of ${{\rm{PF}}}_{6}^{-}$ in the first solvation shell of a Li^+^ ion is much longer than EC and DMC on both short and long time scales, we find that the rotational diffusion constant ${D}_{R}^{{{\rm{PF}}}_{6}^{-}}$ of ${{\rm{PF}}}_{6}^{-}$ is surprisingly larger than ${D}_{R}^{{\rm{EC}}}$ and ${D}_{R}^{{\rm{DMC}}}$, indicating the faster rotational dynamics of ${{\rm{PF}}}_{6}^{-}$ than EC and DMC. This fast rotation of a ${{\rm{PF}}}_{6}^{-}$ ion is ascribed to the fact that a ${{\rm{PF}}}_{6}^{-}$ ion has six F atoms and each F atom tends to form a bond with a Li^+^ ion. It causes the reduction in the energy barrier needed to be overcome for the rotation within the solvation shell of a Li^+^ ion. The slow translational motion and fast rotational motion of ${{\rm{PF}}}_{6}^{-}$ indicate that a bonding of one F atom of ${{\rm{PF}}}_{6}^{-}$ with Li^+^ remains for short time and is replaced by one of the other F atoms of the same ${{\rm{PF}}}_{6}^{-}$ ion.

## Conclusion

The lithium solvation structure and dynamics are of great importance to understand lithium ion batteries. It is known that the formation of the SEI on the electrode is significantly affected by the solvation structure of a Li^+^ ion, since the most contribution to the SEI is ascribed to the decomposition of the solvents in the solvation shell of a Li^+^ ion near the electrode^17^. Thus, the information of the primary solvation structure of a Li^+^ ion is critical for the performance of lithium ion batteries and many research has studied the solvation structure in nonaqueous electrolytes with binary or ternary solvents^9,17,46^. In addition, the solvation dynamics can greatly affect the mobility of a Li^+^ ion^15^. The faster exchange dynamics of solvents in the solvation shell invokes the weaker rigidity of the lithium solvation shell. The solvation structure and dynamics are strongly correlated in such a way that the exchange dynamics in the lithium solvation shell is affected by the solvent composition of the shell^27^. However, the relation between the solvation structure and the exchange dynamics is far from being fully understood.

In this work, we have performed molecular dynamics simulations to investigate the dynamical properties of nonaqueous electrolytes as a function of the mixture ratio of binary solvents. We have found that the exchange dynamics of EC and DMC in the lithium solvation shell shows a non-monotonic behavior on a short time scale with respect to χ_EC_. It indicates that the response of the rigidity of the solvation shell to the thermal fluctuation is different according to the composition of the shell and the response does not change monotonically with the increasing number of EC in the solvation shell. We have further found that this non-monotonic behavior on a short time scale keeps preserved on a long time scale. As χ_EC_ increases, the average distances of EC and DMC from a Li^+^ ion increase, so that the two solvents move toward the boundary of the lithium solvation shell. However, the diffusion of both solvents slows down due to the increase in the viscosity. Thus, with a given size of the lithium solvation shell, it seems that the resulting exchange dynamics of two solvents is ascribed to a competition between the location of solvents in the solvation shell and the motion of them. Furthermore, our results show that the binding angle of DMC to a Li^+^ ion seems to be insensitive to a change in χ_EC_, whereas the binding angle of EC gradually decreases upon increasing χ_EC_. The rotational dynamics of EC shows a different dependences in magnitude of *D*_*R*_ on χ_EC_ compared to the rotational dynamics of DMC. We note that a ${{\rm{PF}}}_{6}^{-}$ ion presents many interesting features in structure and dynamics. The average distance from a Li^+^ ion shows a minimum around at χ_EC_ = 30% different from EC and DMC. The rotational dynamics of ${{\rm{PF}}}_{6}^{-}$ is faster than EC and DMC, whereas the translational dynamics of it is the slowest. Finally, we believe that our results will give valuable insights to broaden our understanding of nonaqueous electrolytes of lithium ion batteries.

## Methods

We perform molecular dynamics (MD) simulations of nonaqueous electrolytes of lithium ion batteries consisting of a solution of 1M lithium hexafluorophosphate (LiPF_6_) salt in a binary solvent mixture of ethylene carbonate (EC) and dimethyl carbonate (DMC). We carry out all simulations using the MD simulation package, LAMMPS^47^. We implement the OPLS/AA force field to describe the molecular interaction of the solvents^15,48^. We compute the long-range interactions using particle-particle particle-mesh (PPPM) algorithm. For the non-bonded interaction, we use the Lennard-Jones interaction with a cutoff of 10 Å. We use the combination rule of Lorentz-Berthelot for the intermolecular interactions. We perform the simulations in the *NVT* ensemble, where *N*, *V* and *T* are the number of molecules, the volume, and the temperature, respectively. For salt, we use *N*_salt_ = 176. For solvents, we use *N*_EC_ = 264∼1584 and *N*_DMC_ = 832∼1872 depending on the EC fraction and use *L* = 6.7108 nm as the linear size of the simulation box, which gives the similar density to the experimental density^49^. We keep the temperature constant via the Nóse-Hoover thermostat with a time constant of 0.1 ps during the simulations. We equilibrate the system for *t* = 40 ns and collect the data for additional time *t* = 6 ns for each 1 ps timestep. We apply periodic boundary conditions in all three directions of the simulation box. We use Δ*t* = 1 fs as a timestep of the simulation. We investigate electrolytes in a range of the solvent mixture ratios from EC:DMC = 10%:90% up to 60%:40% (in volume %). We run 25 independent simulations to collect trajectories for improving the statistics. All averages and the sample standard deviations (error bars) in the figures are calculated from 25 independent datasets.

## Data Availability

The datasets generated during and/or analysed during the current study are available from the corresponding author on reasonable request.
